# Experimental and genetic evidence for the impact of *CD5 *and *CD6 *expression and variation in inflammatory bowel disease

**DOI:** 10.3389/fimmu.2022.966184

**Published:** 2022-09-21

**Authors:** Sergi Casadó-Llombart, María Velasco-de Andrés, Cristina Català, Alejandra Leyton-Pereira, Rebeca Gutiérrez-Cózar, Belén Suárez, Noelia Armiger, Esther Carreras, Miriam Esteller, Elena Ricart, Ingrid Ordás, Javier P. Gisbert, María Chaparro, María Esteve, Lucía Márquez, David Busquets, Eva Iglesias, Esther García-Planella, María Dolores Martín-Arranz, Juliane Lohmann, C. Korcan Ayata, Jan Hendrik Niess, Pablo Engel, Julián Panés, Azucena Salas, Eugeni Domènech, Francisco Lozano, ENEIDA Project of GETECCU, Alfredo J. Lucendo

**Affiliations:** ^1^ Immunoreceptors del Sistema Innat i Adaptatiu, Institut d’Investigacions Biomèdiques August Pi i Sunyer (IDIBAPS), Barcelona, Spain; ^2^ Departament de Biomedicina, Facultat de Medicina, Universitat de Barcelona, Barcelona, Spain; ^3^ Servei d’Immunologia, Centre de Diagnòstic Biomèdic, Hospital Clínic de Barcelona, Barcelona, Spain; ^4^ Inflammatory Bowel Disease Group, Institut d’Investigacions Biomèdiques August Pi i Sunyer (IDIBAPS), Barcelona, Spain; ^5^ Inflammatory Bowel Disease Unit, Gastroenterology Department, Hospital Clínic de Barcelona, Barcelona, Spain; ^6^ Centro de Investigación Biomédica en Red de Enfermedades Hepáticas y Digestivas (CIBERehd), Madrid, Spain; ^7^ Gastroenterology Unit, Hospital Universitario de La Princesa, Instituto de Investigación Sanitaria Princesa (IIS-IP), Universidad Autónoma de Madrid (UAM), Madrid, Spain; ^8^ Gastroenterology Department, Hospital Universitari Mútua Terrassa, Terrassa, Spain; ^9^ Gastroenterology Department, Hospital del Mar and Institut Hospital del Mar Investigacions Mèdiques, Barcelona, Spain; ^10^ Department of Gastroenterology, Hospital Universitari de Girona Dr Josep Trueta, Girona, Spain; ^11^ Department of Gastroenterology, Hospital Universitario Reina Sofía, Córdoba, Spain; ^12^ Instituto Maimónides de Investigación Biomédica de Córdoba (IMIBIC), Córdoba, Spain; ^13^ Department of Gastroenterology, Hospital de la Santa Creu i Sant Pau, Barcelona, Spain; ^14^ Department of Gastroenterology, and Innate Immunity Group, IdiPAZ Institute for Health Research, La Paz Hospital, Facultad de Medicina, Universidad Autónoma de Madrid, Madrid, Spain; ^15^ Life & Medical Sciences (LIMES) Institute, University of Bonn, Bonn, Germany; ^16^ Department of Biomedicine, University of Basel, Basel, Switzerland; ^17^ University Center for Gastrointestinal and Liver Diseases, St. Clara Hospital and University Hospital, Basel, Switzerland; ^18^ Gastroenterology Department, Hospital Universitari Germans Trias i Pujol, Badalona, Spain

**Keywords:** Crohn’s disease, inflammatory bowel disease, ulcerative colitis, CD5, CD6

## Abstract

Crohn’s disease (CD) and ulcerative colitis (UC) are inflammatory bowel diseases (IBD) resulting from the interaction of multiple environmental, genetic and immunological factors. *CD5* and *CD6* are paralogs encoding lymphocyte co-receptors involved in fine-tuning intracellular signals delivered upon antigen-specific recognition, microbial pattern recognition and cell adhesion. While *CD5* and *CD6* expression and variation is known to influence some immune-mediated inflammatory disorders, their role in IBD remains unclear. To this end, *Cd5*- and *Cd6*-deficient mice were subjected to dextran sulfate sodium (DSS)-induced colitis, the most widely used experimental animal model of IBD. The two mouse lines showed opposite results regarding body weight loss and disease activity index (DAI) changes following DSS-induced colitis, thus supporting *Cd5* and *Cd6* expression involvement in the pathophysiology of this experimental IBD model. Furthermore, DNA samples from IBD patients of the ENEIDA registry were used to test association of *CD5* (rs2241002 and rs2229177) and *CD6* (rs17824933, rs11230563, and rs12360861) single nucleotide polymorphisms with susceptibility and clinical parameters of CD (n=1352) and UC (n=1013). Generalized linear regression analyses showed association of *CD5* variation with CD ileal location (rs2241002^CC^) and requirement of biological therapies (rs2241002^C^-rs2229177^T^ haplotype), and with poor UC prognosis (rs2241002^T^-rs2229177^T^ haplotype). Regarding *CD6*, association was observed with CD ileal location (rs17824933^G^) and poor prognosis (rs12360861^G^), and with left-sided or extensive UC, and absence of ankylosing spondylitis in IBD (rs17824933^G^). The present experimental and genetic evidence support a role for *CD5* and *CD6* expression and variation in IBD’s clinical manifestations and therapeutic requirements, providing insight into its pathophysiology and broadening the relevance of both immunomodulatory receptors in immune-mediated disorders.

## Introduction

Inflammatory bowel diseases (IBD) are a group of chronic inflammatory conditions of the gastrointestinal tract, including ulcerative colitis (UC) and Crohn’s disease (CD). The precise etiology of IBDs remains unknown, though their relation to multiple and diverse genetic, immunological and environmental factors is accepted. Genome-wide association studies (GWAS) have identified immune-related genes associated to susceptibility and/or clinical manifestations that point to an inappropriate regulation of innate and/or adaptive immune responses in IBD (1). However, these polymorphisms alone do not account for IBD heritability, suggesting that other environmental, epigenetic and genetic factors, including rare variants, must be in place (1).

*CD5* and *CD6* are paralogs sharing homology in tissue expression patterns, structure and function (2–4). They encode signal-transducing surface co-receptors expressed on all T and B1a cells and involved in the fine tuning of intracellular activation signals delivered upon specific antigen recognition by lymphocyte’s clonotypic receptors (5). Both CD5 and CD6 receptors are composed of an extracellular region encompassing three tandem scavenger receptor cysteine-rich (SRCR) domain repeats, a transmembrane region, and a cytoplasmic region devoid of catalytic activity but well adapted for phosphorylation and association with downstream signaling effectors. Importantly, CD5 and CD6 are physically associated with the T cell receptor complex (TCR) with which co-localize at the center of the immunological synapse (6), providing inhibitory (CD5) and activating/inhibitory (CD6) signals (7). This is likely achieved through interaction with endogenous counter-receptors such as CD166/activated leukocyte cell adhesion molecule (ALCAM) (8), Galectins 1 and 3 (9), and CD318/CUB domain-containing protein 1 (CDCP-1) (10) for CD6, and still ill-defined ligands (CD72, IgVh framework, gp200, gp40-80, gp150, IL-6 and CD5 itself) for CD5 (11–18). Both molecules also act as pattern-recognition receptors (PRRs) for microbial-associated molecular patterns (MAMPs), where CD5 interacts with fungal (β-glucan) (19), viral (hepatitis C virus) (20), and parasitic (*E. granulosus* teguments) structures (21), while CD6 does it with bacterial (lipopolysaccharide, lipoteichoic acid and peptidoglycan) (22), viral (gp120 HIV-1) (23), and parasitic (*E. granulosus* tegument) structures (21). This dual role of CD5 and CD6 as both immunomodulatory and microbial PRR receptors is supported by pre-clinical models of infection, autoimmunity and cancer involving *Cd5*- and *Cd6*-deficient mouse lines, as well as infusion of wild-type mice with soluble CD5 and CD6 proteins (24–26).

To date, no *CD5* or *CD6* deficiencies have been reported in humans. However, functionally relevant single nucleotide polymorphisms (SNPs) of *CD5* and *CD6* have been identified, which act as susceptibility or disease modifier markers in autoimmune and neoplastic processes. Allelic combinations of the *CD5* rs2241002 and rs2229177 SNPs resulting in hyper-reactivity to TCR stimulation are associated to more severe systemic lupus erythematosus (SLE) forms, but predict better prognosis in chronic lymphocytic leukemia (CLL) and melanoma (27–29). Moreover, GWAS have involved *CD5* (rs2229177) in rheumatoid arthritis susceptibility (30). Regarding *CD6*, the rs12360861, rs17824933 and rs11230563 SNPs are revealed as disease modifiers in psoriasis, and as susceptibility markers in multiple sclerosis (MS) and Behçet’s disease (31–34). Also, GWAS and meta-analyses have associated the *CD6* rs11230563 SNP to IBD susceptibility (35, 36). However, its role as a disease modifier in IBD, and the involvement of other neighboring SNPs from the *CD6* and *CD5* genes, and from the functionally related *CD166/ALCAM* gene, are still unknown.

The present work explores the consequence of *CD5* and *CD6* expression and variation in experimental and clinical IBD. To this end, we first analyzed the impact of *Cd5* and *Cd6* deficiency on dextran sulphate sodium (DSS)-induced colitis, an experimental model of human IBD (37). Subsequent clinical association studies assessed the impact of *CD5* and *CD6* variations on different clinically relevant manifestations and therapeutic requirements of CD and UC.

## Materials and methods

### Mice

*Cd5*-deficient (*Cd5^-/-^*) mice backcrossed to C57BL/6 background were provided by Chander Raman (University of Alabama at Birmingham) (38). *Cd6*-deficient (*Cd6^-/-^*) C57BL/6 mice were obtained through a development agreement with the Knock-Out Mouse Project Repository (KOMP), an international consortium promoted by the National Institutes of Health (NIH; https://www.komp.org) (39). Wild-type C57BL/6 mice from Charles River Laboratories (France) were bred in our animal facility. All mouse procedures were approved by the Animal Experimentation Ethical Committee from University of Barcelona.

### DSS-induced mouse colitis model

Colitis was induced by administration of 2% (w/v) 36-50 kDa DSS (MP Biomedicals) in drinking water for 5 days to 11- to 19- week-old wild-type, *Cd5^-/-^* and *Cd6^-/-^* female mice of C57BL/6 background weighing >20 g. Body weight and disease activity index (DAI) were monitored every day. DAI was scored as follows: rectal bleeding (absent=0, present=1), animal motility (normal=0, reluctant=1, hunched=2), fur appearance (normal=0, ruffled=1, spiky=2) and body weight loss (none=0, 0-5%=1, 5-10%=2, 10-15%=3, >15%=4). At day 8, mice were euthanized by cervical dislocation for collection of blood and organ samples. Colons were measured and weighted, and terminal pieces were collected for histology and RNA extraction. EDTA-anticoagulated blood was centrifuged in heparinized capillaries for 30 min at 1000 xg and hematocrit was calculated as the length of packed red blood cells (RBC) divided by the total blood length (RBC + serum) multiplied by 100. For RBC count, blood was diluted in PBS and RBC were counted with a hemocytometer. For microbiological analyses, mesenteric lymph nodes (mLN) and liver were collected under sterile conditions and disaggregated through a 40 μm nylon mesh for overnight (o/n) seeding at 37 °C on Columbia agar plates with 5% sheep blood (Becton-Dickinson) and colony forming unit (cfu) counting. Pieces of ~2 mm were cut from the terminal colon of mice and submerged in RNA later (Sigma) o/n at 4 °C before being stored dry at -80 °C for further RNA analysis or fixed in PBS containing 4% paraformaldehyde during 48 h for histological studies. RNA was extracted using the TRIzol^®^ Reagent (Life Technologies) and the PureLink™ RNA Mini Kit (Ambion, Life Technologies) following manufacturer’s instructions, with the aid of a QIAGEN TissueLyser. RNA was quantified and retrotranscribed into cDNA by using the High-capacity cDNA Kit (Life Technologies). Cytokine mRNA levels were assessed by real-time quantitative PCR (RT-qPCR) with the TaqMan™ Fast Universal PCR Master Mix No AmpErase™ UNG (Life Technologies) using a 7900HT fast real-time PCR system (Applied Biosystems, Foster City, CA, US) and the following FAM gene expression assays: Mm01179194_m1 (*Cd3e*), Mm00435532_m1 (*Pdcd1*), Mm00432423_m1 (*Cd79a*), Mm01337324_g1 (*Ncr1*), Mm00447885_m1 (*Klrc1*), Mm00447885_m1 (*Mpo*), Mm01324470_m1 (*Lcn2*), Mm00440502_m1 (*Nos2*), Mm00801778_m1 (*Ifng*), Mm00439619_m1 (*Il17a*), Mm00445259_m1 (*Il4*), Mm00439614_m1 (*Il10*), Mm00444241_m1 (*Il22*), Mm00443260_g1 (*Tnf*), Mm00434228_m1 (*Il1b*), Mm00446190_m1 (*Il6*), Mm01178820_m1 (*Tgfb1*), Mm00441259_g1 (*Ccl3*), and Mm04207460_m1 (*Cxcl1*), Mm00450960_m1 (*Tbx21*), Mm01261022_m1 (*Rorc*), Mm00484683_m1 (*Gata3*) and Mm00475162_m1 (*Foxp3*), all from Thermo Fisher Scientific. Relative cytokine mRNA expression normalized to *Gapdh* (Mm99999915_g1) expression was calculated as 2^-ΔΔCt^, where ΔΔCt = (CT_*Gene* *of* *interest* *sample*_ − CT_*GAPDH* *sample*_) − (*CT*_*Gene* *of* *interest* *basal*_ − CT_*GAPDH* *basal*)_.

For histological analysis, fixed samples were included in paraffin. Three micrometer tissue sections were obtained and stained with hematoxylin-eosin. Histology was scored by two independent evaluators according to the following parameters: degree of inflammation (0-3), goblet cell loss (0-2), abnormal or hyperproliferative crypts (0-3), abscesses (0-1), architectural damage (0-2), transmural damage (0-3). Images were obtained with an Eclipse 50i microscope, using a Pan Fluor 10x/0.30 objective and a Digital Sight DS-5M camera, all from Nikon.

For immunohistochemical analysis, paraffin-embedded 5 μm tissue sections were immersed in xylene and dehydrated in ethanol. After antigen retrieval, tissue sections were blocked with PBS 5% FBS. For myeloperoxidase (MPO) chromogenic immunohistochemistry assay, primary goat anti-mouse MPO polyclonal antibody (R&D Systems) was incubated at 4°C overnight. Then, endogenous peroxidase activity was blocked using PBS 0.3% H_2_O_2_ solution for 10 min at room temperature and peroxidase-labelled rabbit anti-goat IgG secondary antibody (SIGMA) was incubated for 1h at room temperature. Tissue sections were stained using 3,3’-diaminobenzidine (DAB; SIGMA) and then hematoxylin staining was performed following standard protocols. Sections were mounted with DPX and visualized at 20x magnifications using a NIKON e600 microscope.

For CD3ϵ and IgM immunofluorescence assay, endogenous biotin was blocked with the Avidin/Biotin blocking kit SP-2001 (VectorLabs) following manufacturer’s indications. Then, primary antibodies rabbit anti-mouse CD3ϵ (Cell signaling, D4V8L; dilution 1/100) and FITC-labelled goat anti-mouse IgM (Southern Biotech; dilution 1/200) were incubated at 4 °C overnight. Biotin-labelled secondary donkey anti-rabbit IgG antibody (Jackson Immunoresearch, dilution 1/200) was incubated for 1 h at room temperature. Finally, A555-labelled streptavidin (Roche, dilution 1/200) was incubated for 20 min at room temperature and samples were mounted with mounting medium (PBS 80% glycerol). Samples were visualized at 10 and 20x magnifications using a NIKON e600 microscope.

### DNA samples from patients and controls

Genomic DNA samples from CD (n=1352) and UC patients (n=1013) were retrieved from the ENEIDA biobank upon approval from the Spanish Working Group on CD and UC (GETECCU) (40). Control genomic DNA samples from volunteer donors of the Blood and Tissue Bank (BST) of the Generalitat de Catalunya (n=604) were purified by using the MagNA Pure 96 DNA and Viral NA Large Volume Kit (Roche Diagnostics, Rotkreuz, Switzerland) and the High-throughput robotic workstation MagNa Pure 96 (Roche Diagnostics). The study was approved by the Ethical Committee of Clinical Research of the Hospital Clínic de Barcelona.

### SNP genotyping

Genomic DNA samples (20 ng) were subjected to RT-PCR in a LightCycler^®^ 480 Instrument (Roche) using TaqMan Genotyping Master Mix and TaqMan probes for *CD5* (rs2229177, rs2241002), *CD6* (rs12360861, rs17824933, rs11230563), and *CD166/ALCAM* (rs6437585) (all from Thermo Fisher), following manufacturer’s instructions. Genotyping failure rate was lower than 0.02 for all SNPs.

### Definitions

Location (terminal ileum, colon, ileocolon, and upper gastro-intestinal) and behavior (nonstricturing and nonpenetrating, stricturing, and penetrating) of CD were classified according to the Montreal classification (41). For statistical analysis of location, a value of 1 was assigned to patients with colonic disease, 2 to patients with ileocolonic disease and 3 to patients with ileal disease, independently of upper gastro-intestinal tract involvement. Upper gastro-intestinal tract involvement (presence vs. absence) was assessed independently of distal ileal and colonic involvement. For statistical analysis of extent in UC patients, a value of 0 was assigned to patients with ulcerative proctitis (Montreal classification E1) and a value of 1 was assigned to patients with left-sided UC or extensive UC (Montreal classification E2 and E3). Prognosis was calculated as previously described: patients not requiring any immunomodulatory nor surgical treatment during at least 4 years of follow-up from diagnosis were classified as “good prognosis” while patients requiring two or more immunomodulatory treatments and/or two or more abdominal surgeries were described as “poor prognosis” (42).

### Statistical analyses

Statistical analysis in patient/donor cohort studies was performed with R 3.6.0 (R Foundation for Statistical Computing, Vienna, Austria), with the packages ‘SNPassoc’, ‘survival’, and ‘haplo.stats’ available at the Comprehensive R Archive Network (CRAN) repository. The ‘association’ function included in the ‘SNPassoc’ package was used to assess linkage between each SNP and desired clinical variables with generalized linear models. For each analysis, 4 models were generated (codominant, dominant, recessive, log-additive), and the model with lowest Akaike information criterion (AIC) was chosen. Analyzed variables were age of onset (calculated date of diagnosis − date of birth), peripheral arthritis/arthralgia, ankylosing spondylitis, sacroiliitis, sclerosing cholangitis, cutaneous manifestations (pyoderma gangrenosum or erythema nodosum), ocular manifestations (uveitis or iritis), requirement of biological treatments and prognosis. Additionally, location of the disease, presence of stenosis, presence of fistulae and perianal disease were included in the CD cohort and extent of the disease was included in the UC cohort. In all analyses, sex and persistent tobacco consumption were included as co-variants. To generate stenosis-free survival and fistulae-free survival curves, time between enrolment and complication (patients with stenosis or fistulae) or between enrolment and last follow-up (patients without stenosis or fistulae) was calculated. Cox proportional hazards regression was used to assess the linkage between each SNP and stenosis-free survival or fistulae-free survival. Association of SNPs with susceptibility to CD, UC or combined (IBD) was assessed with the ‘association’ function by comparing each cohort with the control cohort. P values were corrected for false discovery rate (FDR) with the ‘p.adjust’ function (*q* values). The ‘haplo.glm’ function included in the ‘haplo.stats’ package was used to assess linkage between haplotypes and binary clinical variables with generalized linear models, and odds ratio (OR) and confidence intervals (CI) for these associations were obtained with the ‘haplo.cc’ function.

In the study of animal models, normality of data was assessed with the D’Agostino & Pearson normality test. When data was normally distributed, differences were assessed by t-tests, otherwise Mann-Whitney tests were performed. In multiple comparisons, P values were corrected for false discovery rate.

## Results

### *Cd5* and *Cd6* deficiency modulate DSS-induced colitis

**Figure 1:**
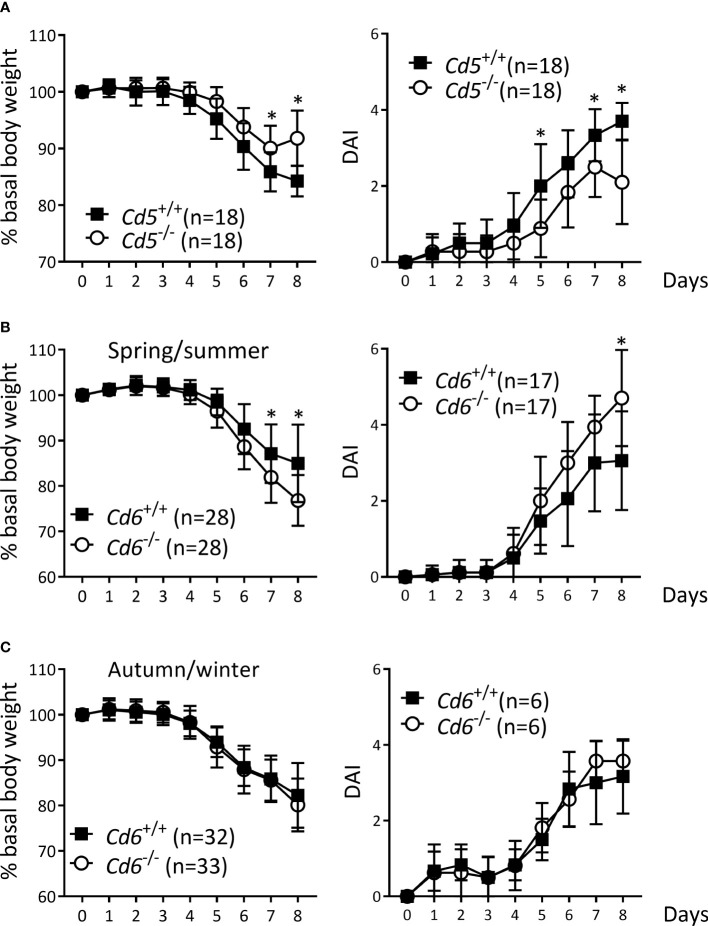
DSS-induced colitis in *Cd5* ^-/-^ and *Cd6* ^-/-^ mice vs. wild-type controls. **(A)** Percentage of basal body weight (left) and DAI (right) of *Cd5* ^-/-^ mice vs. *Cd5* ^+/+^ controls. Data combined from two independent experiments is shown. **(B)** Percentage of basal body weight (left) and DAI (right) of *Cd6* ^-/-^ mice vs. *Cd6* ^+/+^ controls in spring/summer (between April and September). Basal body weight data are combined from four independent experiments, while DAI data are combined from two independent experiments. **(C)** Percentage of basal body weight (left) and DAI (right) of *Cd6* ^-/-^ mice vs. *Cd6* ^+/+^ controls in autumn/winter (between October and February). Basal body weight data are combined from four independent experiments, while DAI data are come from a single experiment. Mean ± SD values are depicted. Statistical differences were assessed by multiple t-tests (one per day) controlled with the FDR approach. *, *q<*0.01.

The putative role of CD5 and CD6 lymphocyte co-receptors in the pathophysiology of IBD was first explored by subjecting *Cd5* ^-/-^ and *Cd6* ^-/-^ mice to the DSS-induced colitis. *Cd5* ^-/-^ mice showed a less aggressive disease than *Cd5* ^+/+^ controls (**Figure 1A**), as deduced from lower body weight loss and DAI, in agreement with a previously published result (43). In contrast, *Cd6* ^-/-^ mice showed an exacerbated phenotype with regard to *Cd6* ^+/+^ controls. Accordingly, *Cd6* ^-/-^ mice presented a higher body weight loss (**Figure 1B**, left) and a higher DAI (**Figure 1B**, right), which was seasonally influenced, as observed in spring/summer and autumn/winter variations (**Figure 1C**).

**Figure 2:**
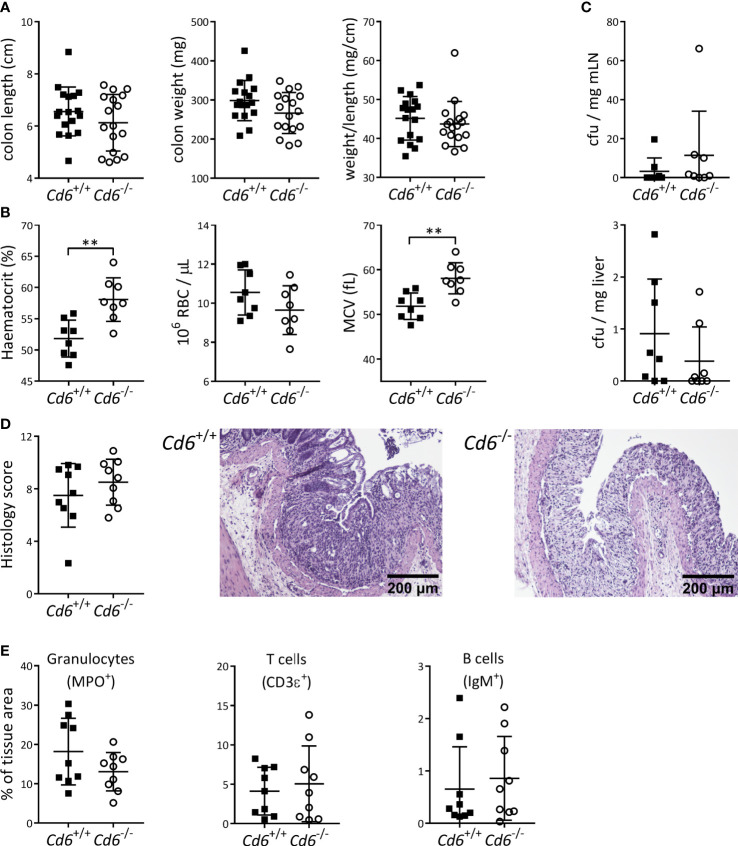
Monitoring of DSS-induced colitis parameters from *Cd6* ^-/-^ mice vs. *Cd6* ^+/+^ controls at day 8 post-induction. **(A)** Dot plot showing colon length, weight and weight to length ratio of *Cd6* ^-/-^ (n=17) and *Cd6* ^+/+^ control (n=17) mice. Mean ± SD values are depicted. Statistical differences were assessed by t-test. **(B)** Hematocrit, RBC count and mean corpuscular volume (MCV) at day 8 from *Cd6* ^-/-^ (n=8) and *Cd6* ^+/+^ (n=8). Mean ± SD values are depicted. Statistical differences were assessed by t-test. **, *p<*0.01. **(C)** Analysis of microbial translocation into mesenteric lymph nodes (mLN; top) and liver (bottom) from the same mice as in **(B)** Depicted are mean ± SD of cfu/mg. Statistical differences were assessed by Mann-Whitney tests. **(D)** Histology score (mean ± SD, left) and representative haematoxylin-eosin stains from DSS-treated *Cd6* ^+/+^ (center) and *Cd6* ^-/-^ (right) mice. Scale bar: 200 μm. Statistical differences were assessed by t-test. **(E)** Immunohistochemical analyses of the terminal colon in DSS-treated mice. Percentage of MPO, CD3ϵ and IgM-stained tissue (mean ± SD) from colon sections. Statistical differences were assessed by t-test.

**Figure 3:**
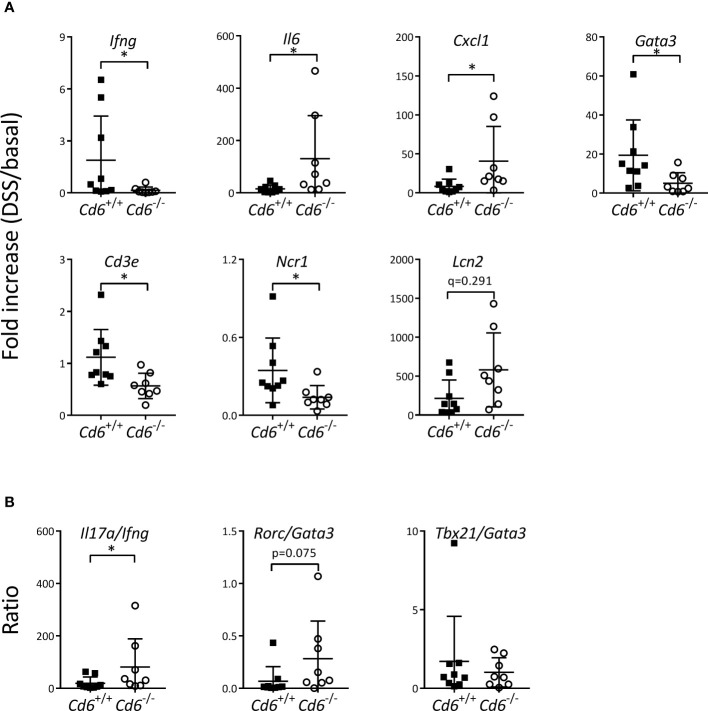
mRNA expression in colons from *Cd6* ^-/-^ vs. *Cd6* ^+/+^ mice at day 8 post DSS-induced colitis. **(A)** Relative mRNA expression of different transcripts from colon samples. Depicted are mean ± SD of mRNA fold increase (DSS/basal). **(B)** Fold increase ratio of indicated mRNA transcripts from colon samples. Ratios were calculated by dividing the fold increase of the following transcripts: *Il17a*, *Ifng*, *Rorc*, *Gata3*, *Tbx21* and *Gata3*. Statistical differences were assessed by Mann-Whitney tests and corrected for FDR. *, *q*< 0.1.

Contrary to the *Cd5* ^-/-^ case, the lack of published information of *Cd6* ^-/-^ mice on the DSS-induced colitis model encouraged a deeper evaluation of different experimental parameters at the end of disease follow-up (day 8). No differences were observed in colon length, weight or weight/length ratio relative to *Cd6* ^+/+^ controls (**Figure 2A**). As illustrated in **Figure 2B**, *Cd6* ^-/-^ mice presented increased hematocrit consistent with higher diarrhea-induced fluid loss, and a trend to lower RBC counts together with increased mean corpuscular volume (MCV) consistent with moderate rectal bleeding and erythroblast production, respectively (44). No differences in cfu count were observed in mLN and liver (**Figure 2C**), arguing against differential bacterial translocation to draining organs as responsible for the differences observed in disease severity. Histological analyses showed noticeable crypt architectural distortion in colon samples from both *Cd6* ^+/+^ and *Cd6* ^-/-^ mice, with no significant differences between their histology scores (**Figure 2D**). Immunohistochemical analyses of the colonic mucosa composition revealed no significant differences in terms of granulocyte (MPO^+^), T cell (CD3ϵ^+^) and B cell (IgM^+^) infiltrates (**Figure 2E**). Gene expression analyses of a wide panel of pro-/anti-inflammatory cytokine and chemokine and transcription factors revealed decreased expression of *Ifng*, *Cd3e*, *Ncr1* and *Gata3* together with increased expression of *Il6* and *Cxcl1* in *Cd6* ^-/-^ mice with regard to controls (**Figure 3A**). A trend towards increased expression of *Lcn2* was also observed (**Figure 3A**). No differences were observed regarding expression of *Tgfb1*, *Tnf*, *Il1b*, *Il17a*, *Il10*, *Il22*, *Tbx21*, *Rorc*, *Foxp3*, *Ccl3*, *Klrc1*, *Pdcd1*, *Cd79a*, *Mpo* and *Nos2* (**Supplementary Figure 1**). Expression of *Il4*, a target of GATA3, was also analyzed but it was undetectable in a high proportion of samples. Additional analyses showed significantly increased *Il17a*/*Ifng* ratio and a similar trend for *Rorc*/*Gata3* but no differences in the *Tbx21*/*Gata3* ratio in *Cd6* ^-/-^ mice (**Figure 3B**).

### *CD5* and *CD6* variants impact clinical expression of IBD

CD (n=1352) and UC (n=1013) patients from the ENEIDA registry and volunteer blood donor controls (n=604) were genotyped for functionally relevant *CD5* (rs2229177, rs2241002), *CD6* (rs12360861, rs11230563, rs17824933), and *CD166/ALCAM* (rs6437585) SNPs. All SNPs were in Hardy-Weinberg equilibrium, except for the rs2241002 in the CD cohort (*p*=0.0276). Description of the study SNPs and cohorts are shown in **Tables 1**, **2**.

**Table 1:** Summary of the *CD5*, *CD6* and *CD166/ALCAM* SNPs analyzed in the present study.

Gene	SNP ID	Location	Major/Minor allele	Effect
*CD5*	rs2241002	Exon 5	C>T	Pro224>Leu
	rs2229177	Exon 10	C>T	Ala471>Val
*CD6*	rs17824933	Intron 1	C>G	CD6Δd3
	rs11230563	Exon 4	C>G	Arg225>Trp
	rs12360861	Exon 5	G>A	Ala271>Thr
*CD166/ALCAM*	rs6437585	5’ UTR	C>T	↑Transcription

**Table 2:** Clinical characteristics of the study cohorts.

Parameter	CD (n=1352)	UC (n=1013)	IBD (n=2365)
Sex			
Male	661 (48.9%)	530 (52.3%)	1191 (50.3%)
Female	691 (51.1%)	483 (47.7%)	1174 (49.6%)
Ethnicity			
Caucasian	1173	856	2029
Arab	13	10	23
Asian	6	3	9
African	5	3	8
Jew	4	1	5
Romani	3	3	6
Other	11	6	17
Smoking*	380 (28.1%)	108 (10.7%)	488 (20.6%)
Age at diagnosis (years)	29.7 (22.4, 41.2)	35.2 (26.8, 47.8)	32.0 (23.7, 44.2)
Follow-up (years)	12.0 (7.4, 19.2)	12.4 (7.4, 19.2)	12.2 (7.4, 19.2)
Extra-intestinal manifestations			
Peripheral arthritis			287 (12.1%)
Ankylosing spondylitis			73 (3.1%)
Sacroiliitis			68 (2.9%)
Sclerosing cholangitis			22 (0.9%)
Cutaneous			158 (6.7%)
Ocular			56 (2.4%)
Location			
Colonic	234 (17.3%)		
Ileocolonic	631 (46.7%)		
Ileal	355 (26.3%)		
Phenotype			
Stricturing	342 (25.3%)		
Penetrating	251 (18.6%)		
Perianal disease	361 (26.7%)		
Extent			
Proctitis		153 (15.1%)	
Left or extensive colitis		828 (81.7%)	
Biological treatments	809 (59.8%)	282 (27.8%)	1091 (46.1%)
Prognosis			
Good	137 (10.1%)	441 (43.5%)	578 (24.4%)
Poor	577 (42.7%)	232 (22.9%)	809 (34.2%)

(*) persistent habit at the last follow-up.

Number of patients is shown for categorical parameters. Median and interquartile range is shown for numerical parameters.

No association was found for any of the SNPs analyzed with disease susceptibility following comparisons of controls with the CD and UC cohorts, either separately (CD vs. controls, UC vs. controls) or together (IBD vs. controls). Next, the effect of the *CD5*, *CD6* and *CD166/ALCAM* gene variants on different clinically relevant parameters of CD (age at diagnosis, behavior, location, perianal disease and prognosis) and UC (age at diagnosis, extent and prognosis) was assessed. A significant association was found for the *CD5* rs2241002^CC^ genotype with preferential ileal location in the CD cohort (**Table 3**). Because CD location can influence the risk of developing stenosis and fistulae, association between the SNP and stenosis-free and fistulae-free survival was tested, but no significant results were found. Association between *CD5* SNPs and upper-gastrointestinal (GI) tract affectation was also not significant. Haplotypic analyses showed increased need of biologic therapies in CD patients carrying the *CD5* rs2241002^C^ rs2229177^T^ haplotype compared with those carrying the most common rs2241002^C^ rs2229177^C^ haplotype (**Table 4**). Similarly, UC patients carrying the *CD5* rs2241002^T^ rs2229177^T^ haplotype had a worse prognosis than those carrying the rs2241002^C^ rs2229177^C^ haplotype (**Table 4**).

**Table 3:** Linear regression analysis of *CD5* rs2241002 and *CD6* rs17824933 SNPs association with CD location. Corrected for sex and smoking.

SNP	Model	Genotype	Colonic =1	Ileo-colonic =2	Ileal =3	mean	s. e.	Difference of means (95% CI)	*q* value
*CD5* rs2241002	Dominant	C/C C/T-T/T	119 103	391 206	228 105	2.148 2.005	0.025 0.035	-0.142 (-0.224,-0.059)	0.005
*CD6* rs17824933	Recessive	C/C-C/G G/G	215 8	557 38	302 31	2.081 2.299	0.021 0.074	0.218 (0.060, 0.377)	0.022

Variable “location” is codified as: colonic=1, ileocolonic=2, ileal=3. p value corrected for FDR. s. e.: standard error.

**Table 4:** Logistic regression analysis of *CD5* haplotype association with biological therapy requirement in CD (top half) and to prognosis in UC (bottom half).

Haplotype		% in CD patients	Biological		*p* value	OR (95% CI)
rs2241002	rs2229177		% no	% yes		
C	C	43.4	45.3	42.2		
C	T	35.9	33.4	37.5	0.048	1.20 (1.00, 1.44)
T	T	17.0	17.4	16.7	0.811	1.02 (0.83, 1.27)
T	C	3.7	3.8	3.7	0.861	1.05 (0.64, 1.72)
**Haplotype**		**% in UC patients**	**Prognosis**		***p* value**	**OR (95% CI)**
**rs2241002**	**rs2229177**		**% good**	**% poor**		
C	C	43.3	45.2	39.5		
C	T	37.1	37.1	37.2	0.345	1.14 (0.87, 1.49)
T	T	14.9	13.7	17.0	0.048	1.42 (1.00, 2.02)
T	C	4.7	3.9	6.3	0.097	1.78 (0.90, 3.51)

**Figure 4:**
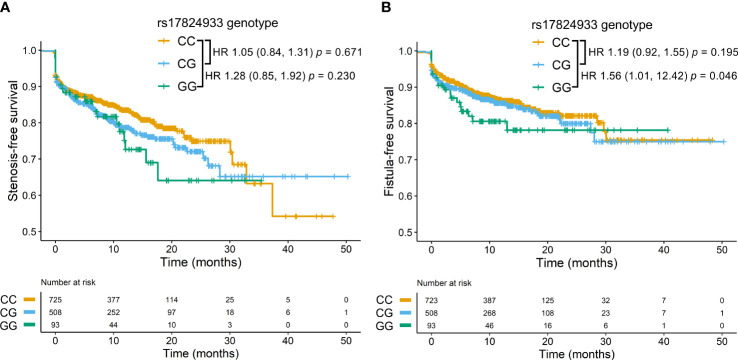
Stenosis and fistulae in CD patients according to rs17824933. Stenosis-free survival **(A)** and fistulae-free survival **(B)** of CD patients carrying different *CD6* rs17824933 genotypes. Statistical differences were assessed by the Cox proportional hazards model. **(A)** In the stenosis-free survival analysis hazard ratio (HR) comparing GG and CC genotypes was 1.28, (95% CI 0.85–1.92), *p* = 0.230, and HR comparing CG and CC genotypes was 1.05, (95% CI 0.84–1.31), *p* = 0.671. **(B)** In the fistulae-free survival analysis HR comparing GG and CC genotypes was 1.56, (95% CI 1.01–12.42), *p* = 0.046, and HR comparing CG and CC genotypes was 1.19, (95% CI 0.92–1.55), *p* = 0.195.

Regarding *CD6* SNPs associations, the rs17824933^GG^ genotype was associated with preferential ileal location in CD patients (**Table 3**). This led us to also test association between this SNP and stenosis-free and fistulae-free survival. As seen in **Figure 4**, the *CD6* rs17824933^GG^ genotype was significantly associated with shorter fistula-free survival (HR = 1.56, 95% CI 1.01–12.42, *p* = 0.046). No significant association was found between *CD6* SNPs and upper-GI tract affectation. The *CD6* rs17824933^GG^ genotype was also associated with higher extent (left or extensive colitis) in UC patients (**Table 5**). The *CD6* minor rs12360861^A^ allele showed association with better prognosis in CD patients (**Table 5**). Regarding the appearance of extra-intestinal manifestations, logistic regression analyses showed a significant association of homo- or heterozygous combinations of the *CD6* rs17824933^G^ allele with lower risk of ankylosing spondylitis in the whole cohort of IBD patients (**Table 5**).

**Table 5:** Logistic regression analysis of *CD6* SNP association with CD prognosis (top), UC extent (middle), and ankylosing spondylitis in IBD patients (bottom), corrected for sex and smoking.

SNP	Model	Genotype	Good prognosis (%)	Poor prognosis (%)	OR (95% CI)	*q* value
rs12360861	Log-additive	A alleles (0, 1, 2)	127 (18.9)	544 (81.1)	0.62 (0.45, 0.86)	0.027
**SNP**	**Model**	**Genotype**	**Proctitis (%)**	**Left/extensive colitis (%)**	**OR (95% CI)**	***q* value**
rs17824933	Recessive	C/C-C/G G/G	151 (98.7) 2 (1.3)	758 (93.0) 57 (7.0)	5.68 (1.37, 23.48)	0.010
**SNP**	**Model**	**Genotype**	**No ankylosing spondylitis (%)**	**Ankylosing spondylitis (%)**	**OR (95% CI)**	***q* value**
rs17824933	Dominant	C/C C/G-G/G	456 (54.1) 387 (45.9)	51 (71.8) 20 (28.2)	0.45 (0.27, 0.78)	0.016

No statistical association was observed with any of the clinical parameters analyzed for *CD166/ALCAM* rs6437585 SNP, which has been reported to influence *CD166/ALCAM* transcriptional activity and MS risk (45, 46).

## Discussion

We provide experimental and clinical evidence for the involvement of CD5 and CD6 expression and variation in IBD. Previous GWAS and meta-analysis studies have identified the CD6 locus (SNP rs11230563) as a susceptibility marker in CD and UC, thus supporting its contribution to IBD etiopathogenesis (35, 36). Here, we used genetically modified mice and candidate gene-driven association analyses to clinical traits and prognosis with functionally relevant SNPs from the *CD5* and *CD6* paralogs, as well as from *CD166/ALCAM*.

Etiopathogenic factors for IBD include host genetic susceptibility, dysregulated immune response, intestinal dysbiosis, and impairment of intestinal epithelial barrier function. Under normal circumstances, there is continuous crosstalk between gut microbiota and the immune system, where gut microbiota modulates the host’s innate and adaptive immunity and vice versa (47). Gut microbiota is in close contact with the intestinal barrier, consisting of an epithelial cell layer and a variety of immune cells of hematopoietic origin. Cells from the intestinal barrier (both epithelial and hematopoietic) sense and signal the presence of microbial components *via* PRRs, which belong to different structural families such as lectin C-type, leucine-rich repeats (LRR), immunoglobulin (Ig), or scavenger receptor cysteine-rich (SRCR) domains (48). Both CD5 and CD6 are lymphocytic members of the SRCR superfamily, expressed by all T cells and the B1a subset responsible for production of polyreactive natural IgM antibodies (4, 49). CD5 and CD6 are also represented in certain immune cells subsets present in mucosal barriers such as regulatory T (Treg) and B (B1a, Breg) cells, certain macrophage and dendritic cells and innate lymphoid cells (NK, iNKT, ILCs) (50–52). CD166/ALCAM, the best characterized CD6 ligand, is also found in the gastrointestinal epithelial tract (53). Increased expression of both CD6 and CD166/ALCAM has been reported in inflamed mucosa from IBD patients, a fact that is attributed to higher CD6-expressing T cell infiltration rather than surface CD6 expression levels (54). This may relate to quantitative trait loci studies in which the rs11230584 SNP in the intergenic region between *CD5* and *CD6* modulates expression of both genes in IBD patients but not in healthy controls (55). Taken together, their tissue and cell expression pattern, microbial recognition properties and ability to modulate lymphocyte activation/differentiation and cell adhesion provide the basis for considering both CD5 and CD6 as contributors to IBD pathogenesis.

The observation that both CD5- and CD6-deficient mice differ in their response to DSS-induced colitis further supports their involvement in IBD. In *Cd5* ^-/-^ mice, attenuated DSS-induced colitis was observed in agreement with a previous report (43). The mechanism underlying such attenuated colitis has already been explored and attributed to increased suppressive function of Treg cells from *Cd5* ^-/-^ mice (43), a fact that was not confirmed by others (56). An alternative mechanism could be the increased activation-induced cell death (AICD) in *Cd5* ^-/-^ effector T cells as a result of inhibitory role assigned to the CD5 receptor (7, 57). Further evidence for CD5 expression involvement in IBD comes from recent report showing that inducible *Cd5*-deficient mice in the autoimmune-prone non-obese diabetic (NOD) background undergo exacerbated DSS-induced colitis by modifying T cell effector function (58).

Regarding *Cd6* ^-/-^ mice, no analysis of DSS-induced colitis has been brought forward, in spite of reports of *Cd6* ^-/-^ mice behavior in several other immune-related inflammatory disease models (i.e., intestinal ischemia-reperfusion, bovine or avian type II collagen-induced arthritis, chronic graft-versus-host disease-induced lupus-like, imiquimod-induced psoriasis-like skin inflammation, experimental autoimmune encephalitis, and autoimmune uveitis) (10, 34, 39, 59–62). CD6 deficiency results in attenuated or exacerbated phenotypes according to mouse background and experimental models responsive to different underlying mechanisms (e.g., increased AICD or defective Treg function). This puzzling situation has been unveiled by CD6 receptor’s multitask signalosome with opposite functions in T cell activation (7). CD6 multifaceted role accounts for past difficulties in classifying it as a co-inhibitory or -stimulatory receptor.

Here we observed that *Cd6* ^-/-^ mice exhibit an increased body weight loss and DAI upon DSS-colitis induction, in conjunction with differential expression of certain mRNA transcripts. This included decreased expression of *Ifng* —the prototypical Th1 cytokine— and *Gata3* —the master regulator of Th2 differentiation—, no differences in *Il17a* and *Il10* expression, and increased expression of *Il6* and *Cxcl1* —a cytokine and a chemokine involved in the Th17 function, all this pointing to a somehow misbalanced Th1/Th2/Th17 response. Additionally, reduced mRNA expression of *Ncr1* —coding for NKp46, one of the NK triggering receptors— and a trend towards increased *Lcn2* —coding for lipocalin-2, also named NGAL, a neutrophil secondary granule marker— in colons of *Cd6* ^-/-^ mice undergoing DSS-induced colitis was observed. These findings would fit with the observation that *i*) decreased NK cell activity would lead to increased granulocyte infiltrate in DSS-induced colitis (63), and *ii*) increased lipocalin-2 expression would act as marker and a counter reactor of colonic inflammation (64, 65).

Given the multifaceted nature of CD6, the above-mentioned mechanistic findings for exacerbated symptoms during DSS colitis in *Cd6* ^-/-^ mice do not exclude other possibilities such as decreased Treg functionality in *Cd6* ^-/-^ mice (59), a cell subset known for its role in mucosal protection during DSS-induced colitis (66). Another possibility could be the deficient production of natural antibodies, as found in *Cd6* ^-/-^ mice from DBA-1 background (60), and confirmed by us in the C57BL/6 background *Cd6* ^-/-^ mice used here (67). Natural antibodies are an innate component of humoral immunity and have a protective role in IBD (68, 69).

In humans, no *CD5* or *CD6* deficiencies have been reported. Functionally relevant *CD5* or *CD6* SNPs previously described act as susceptibility or disease modifier markers for immune-related disorders. Regarding *CD5*, the rs2241002 and rs2229177 SNPs cause nonsynonymous substitutions at the extracellular (Pro224>Leu) and cytoplasmic (Ala471>Val) regions, respectively, which are relevant to CD5-mediated signal transduction (70). Thus, homozygous carriers of the ancestral Pro224-Ala471 (rs2241002^C^ and rs2229177^C^) *CD5* haplotype are hyper-reactive to TCR/CD3 cross-linking, and present more severe clinical forms of SLE (27) but better CLL and melanoma prognosis (28, 29). Regarding the functionality of *CD6* SNPs, the intronic rs17824933^G^ allele identified as a susceptibility marker for MS causes over-expression of a CD6 isoform devoid of the CD166/ALCAM-binding domain (CD6Δd3) concomitant with diminished proliferation and long-term activation of CD4^+^ T cells (71, 72). Also, the MS-protective haplotype involving the *CD6* rs11230563^C^ and rs2074225^C^ SNPs results in higher surface CD6 expression on several lymphocyte subsets (CD4^+^ and CD8^+^ naïve T, and NKT cells) (31).

In our genetic analysis the *CD5* rs2241002 SNP, which causes a nonsynonymous substitution in the extracellular SRCR domain 2 of CD5 (Pro224>Leu), showed association with CD location. Further analyses showed association of *CD5* haplotypes containing the cytoplasmic rs2229177^T^ variant with severity parameters in CD (requirement of biological treatments) and UC (poor prognosis) patients. The rs2229177^T^ variant involves the substitution of ancestral Ala471 for Val, which results in increased CD5 inhibitory capacity (27, 70). This can turn activated lymphocytes less sensitive to AICD and more damaging, thus making more intensive therapies necessary.

Analysis of *CD6* SNPs showed association of the rs17824933^G^ allele with preferred ileal CD location and increased UC extent. These results consolidate the damaging effect of the rs17824933^G^ allele in inflammatory diseases, as suggested from its association with more aggressive forms of psoriasis and with MS susceptibility (34, 46). Patients with left-sided or extensive UC also tend to need more aggressive therapies and are at higher risk of developing colorectal cancer (73). A relatively short follow-up (median 12.37 years; Q1 7.43 years; Q3 19.21 years) may underlie the lack of significant differences observed for this SNP regarding prognosis (**Supplementary Table 1**).

The *CD6* rs17824933^G^ allele was further associated with lower risk of ankylosing spondylitis in the whole IBD cohort. This result appears to contradict the above-mentioned deleterious contribution of this variant in UC, as well as in psoriasis and MS. However, this variant also showed association with a more ileal location of CD. Articular extra-intestinal manifestations of IBD are more common in patients with colonic disease than in those with small-bowel disease (74). Thus, preferential ileal location in CD patients may account for the association of rs17824933^G^ with lower ankylosing spondylitis risk.

The study also showed association of the *CD6* rs12360861 SNP with prognosis in CD patients but not susceptibility, in agreement with a major genetic contribution to prognosis from loci distinct from those driving disease susceptibility, applicable in this case (42).

As stated above, genetic susceptibility is only one of the known factors in IBD etiopathogenesis. The importance played by other environmental factors is illustrated by seasonal onset and exacerbation patterns in IBD patients (75). We have observed seasonal variations also for *Cd6* ^-/-^ mice regarding susceptibility to DSS-induced colitis. More precisely, the exacerbated DSS-induced colitis phenotype of *Cd6* ^-/-^ mice manifested during the spring/summer but not the autumn/winter season (**Figure 1C**), reminiscent of other mouse models of human diseases (i.e., EAE) (76). Though incompletely understood, it has been proposed that seasonal variations might be regulated by endogenous circannual rhythms, since they are found even when the animals are subjected to a constant, controlled environment, and genetically regulated (77). Seasonality in *Cd5* ^-/-^ mice could be neither confirmed nor denied, since the two experiments performed were carried out in the summer season (July and September). However, recent unpublished results from a collaborative study show seasonality phenomena in Cd5^-/-^ mice upon mannan-induced psoriatic arthritis induction (Merino R and Merino J, University of Cantabria, Spain).

The main strengths of our study are the use of a large patient cohort, which together with the experimental mouse model highlights a role for *CD5* and *CD6* in IBD. We also acknowledge some caveats in our study. Separate breeding of *Cd5* ^-/-^ or *Cd6* ^-/-^ mice and their wild-type counterparts can be a source of confusion, which we minimized by periodic colony refresh, the use of high sample sizes, and a large number of repetitions. We measured mRNA expression as a proxy for protein expression, but correlation between mRNA and protein levels is limited. Therefore, further protein expression assays (e.g.: flow cytometry) will be needed to ascertain the molecular mechanisms underlying differences between *Cd6* ^-/-^ and their wild-type counterparts. Similarly, further molecular mechanisms driving clinical SNP associations in IBD patients are to be identified.

In conclusion, our findings support a role for the CD5 and CD6 lymphocyte receptors in the pathophysiology of IBD and hint at their potential in patient stratification and as therapeutic targets. The latter is particularly valid for CD6, where the humanized anti-CD6 mAb Itolizumab currently represents a therapeutic option in several immune-mediated disorders (78), since could modulate the activity of the T cell subsets (i.e., Th1 and Th17) involved in their pathogeny (79).

## Data availability statement

The original contributions presented in the study are included in the article/**Supplementary Material**. Further inquiries can be directed to the corresponding author/s

## Ethics statement

The studies involving human participants were reviewed and approved by Ethical Committee of Clinical Research of the Hospital Clínic de Barcelona. The patients/participants provided their written informed consent to participate in this study. The animal study was reviewed and approved by Comitè Ètic d’Experimentació Animal, Universitat de Barcelona, Spain.

## Contributing GETECCU members

Alfredo J. Lucendo, Gastroenterology Department, Hospital General de Tomelloso, IIS-IP, Ciudad Real, Spain, and Centro de Investigación Biomédica en Red de Enfermedades Hepáticas y Digestivas (CIBERehd). Jordi Guardiola, Hospital Universitari de Bellvitge (IDIBELL), l’Hospitalet de Llobregat, Spain. Xavier Calvet, Servei d’Aparell Digestiu, Hospital Universitari Parc Taulí, Departament de Medicina, Universitat Autònoma de Barcelona, Sabadell, Spain, and CIBERehd. Lorenzo Oliván, Hospital San Jorge, Huesca, Spain. Marta Piqueras, Gastroenterology Department, Consorci Sanitari de Terrassa, Barcelona, Spain.

## Author contributions

Conceptualization: FL, AS, SC-L, and JP. Mouse studies: SC-L, MV-dA, CC, NA, AL-P, RG-C, EC, MiE, JL, KA, JHN, PE, and AS. Genetic studies: SC-L, MV-dA, CC, NA, AL-P, BS, EC, and JL. Sample and clinical information collection: ER, IO, JPG, MaE, LM, DB, EI, EG-P, MDM-A, AJL, JG, XC, LO, MP, and JP. Statistical analyses and figures: SC-L, EC, and JL. Writing original draft: SC-L and FL. All authors read, critically revised and approved the final version of the manuscript.

## Funding

This work was supported by Spanish Ministerio de Economía y Competitividad (MINECO, SAF2016-80535-R) and Ministerio de Ciencia e Innovación (MCIN/AEI/10.13039/501100011033, PID2019-106658RB-I00), co-financed by European Development Regional Fund “A way to achieve Europe” ERDF, and Agència de Gestió d’Ajuts Universitaris i de Recerca from Generalitat de Catalunya (2017/SGR/1582). SC-L, MV-dA, CC, AL-P, and EC are recipients of fellowships from Spanish Ministerio de Educación, Cultura y Deporte (FPU15/02897), Spanish MINECO (BES-2014-069237 and BES-2017-082107), Chilean Agencia Nacional de Investigación y Desarrollo (2018-72190154), and European Community Seventh Framework Program (FP7/2007/2013; 229673), respectively. SC-L and JL are recipients of short-term fellowships from European Federation of Immunological Societies-Immunology Letters (EFIS-IL) and Erasmus+ from the European Union, respectively. The ENEIDA registry of GETECCU is supported by Biogen, Janssen, Takeda and Pfizer. The funders were not involved in the study design, collection, analysis, interpretation of data, the writing of this article or the decision to submit it for publication.

## Acknowledgments

We thank Belchin Kostov for statistical analysis support, Marcos Isamat for manuscript editing and critical comments, and Silvia Ariño for technical help with RT-PCR and immunohistochemistry quantification and interpretation. We are indebted to GETECCU and the IDIBAPS Biobank for clinical data and sample procurement.

## Conflict of interest

FL is founder and ad-honorem scientific advisor of Sepsia Therapeutics.

The remaining authors declare that the research was conducted in the absence of any commercial or financial relationships that could be construed as a potential conflict of interest.

## Publisher’s note

All claims expressed in this article are solely those of the authors and do not necessarily represent those of their affiliated organizations, or those of the publisher, the editors and the reviewers. Any product that may be evaluated in this article, or claim that may be made by its manufacturer, is not guaranteed or endorsed by the publisher.
