# Supplementary material for: Experimental and genetic evidence for the impact of CD5 and CD6 expression and variation in inflammatory bowel disease
Source: Front Immunol. 2022 Sep 21;13:966184. doi: 10.3389/fimmu.2022.966184 (PMC9532939; doi:10.3389/fimmu.2022.966184)
Supplement: Supplementary file 1 [file DataSheet_1.pdf]

Supplementary Material

1 Supplementary Figure 1

mRNA expression in colons from *Cd6*$^{-/-}$ *vs.* *Cd6*$^{+/+}$ mice at day 8 post DSS-induced colitis. Relative mRNA expression of different transcripts from colon samples. Depicted are mean ± SD of mRNA fold increase (DSS/basal).

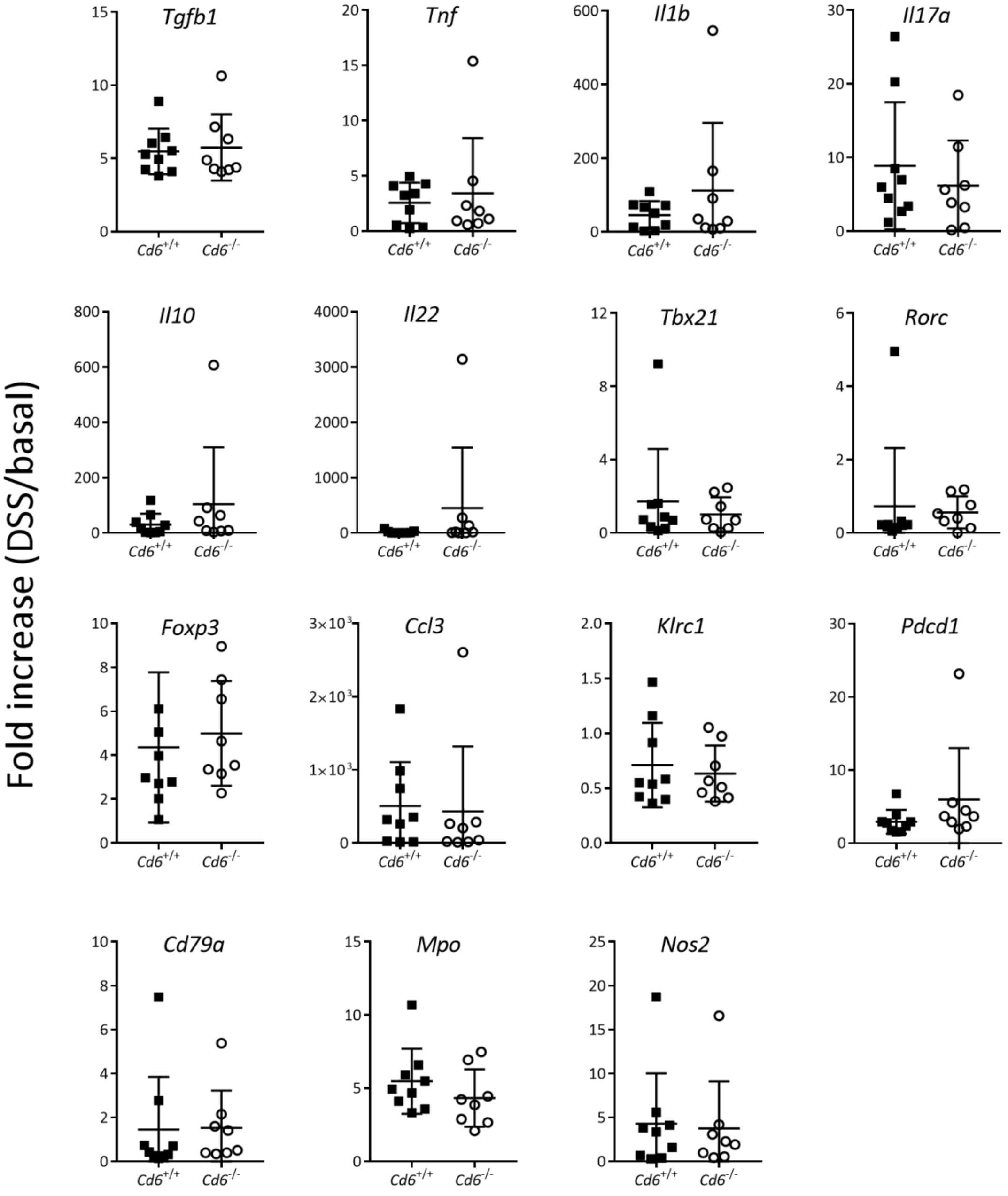

2 Supplementary Table 1

Logistic regression analysis of *CD6* rs17824933 SNP association with UC prognosis. Corrected for sex and smoking.

SNP	Model	Genotype	Good prognosis (%)	Poor prognosis (%)	OR (95% CI)	*q* value
rs17824933	Recessive	C/C-C/G	146 (92.4)	650 (93.9)		0.586
		G/G	12 (7.6)	42 (6.1)	0.79 (0.40, 1.53)	
